# Functional Movement Screen Scores Are Comparable in Volleyball Players with and Without Back Pain—The FMS-VBP Study

**DOI:** 10.3390/jcm14186502

**Published:** 2025-09-15

**Authors:** Tomasz Chomiuk, Przemysław Kasiak, Andrzej Filipek, Artur Mamcarz, Daniel Śliż

**Affiliations:** 1 3rd Department of Internal Medicine and Cardiology, Medical University of Warsaw, 02-097 Warsaw, Poland; 2 Faculty of Health Sciences, Medical University of Warsaw, 02-097 Warsaw, Poland

**Keywords:** volleyball, athlete, FMS, functional movement score, back pain

## Abstract

**Background/Objectives**: Volleyball is one of the most common sports that require dynamic movements. Back pain is one of the most prevalent medical conditions. The Functional Movement Screen (FMS) is used to analyze motor skills, assess movement quality, and target preventive strategies. Therefore, we analyzed the differences in FMS scores among volleyball players with and without back pain. **Methods**: We recruited 79 athletes (23 [29.1%] females; age 28.8 ± 6.4 years, BMI 24.5 ± 3.0 kg/m^2^). Participants were required to be at least 18 years old and have at least 1 year of experience in training for volleyball. We stratified the population between individuals with and without back pain. All the subjects performed the FMS, which consisted of seven exercises. Participants with back pain additionally completed the Oswestry Disability Index (ODI) questionnaire. **Results**: In total, 31 (39.2%) athletes reported that they currently experienced back pain, and 67 (84.8%) reported that they had ever experienced back pain regardless of the degree. The population achieved an ODI score of 3.9 ± 6.1. In total, 20 (66.7%) participants rated their back pain as mild, and only 1 (3.2%) athlete graded back pain as severe. There were no significant differences in each exercise during the FMS between males with and without back pain (*p* = 0.07–0.97; *p*_adjusted_ = 0.56–1.00) or females without and with back pain (*p* = 0.30–0.90). Back pain did not influence total FMS scores in males (t(54) = 1.72, *p* = 0.09, *p*_adjusted_ = 0.72) and females (t(21) = −0.09, *p* = 0.93, *p*_adjusted_ = 1.00). **Conclusions**: Back pain is a common medical condition among volleyball players and most often has a mild course. Despite no functional score differences, the high prevalence of back pain suggests the need for prevention.

## 1. Introduction

Back pain is a growing problem in current society [1,2]. Approximately 60–90% of people have experienced this pain at least once in their lives, and forecasts indicate that this percentage may increase further [3]. Studies show that the first symptoms of low back pain appear as early as in teenagers [4]. Back pain is the most common reason for medical appointments in primary care and one of the most common medical conditions overall [5,6].

Athletes also suffer from back pain, especially in the lumbar area [7,8]. It is well known that regular physical activity alleviates the risk of back pain [9]. However, dynamic movements, past injuries, and improper lifestyle habits make back pain a common problem among athletes [10]. It is especially visible among recreational and amateur athletes who cope most with daily life challenges and practice sport simultaneously [11]. Back pain during exercises requires compensational movements and raises the risk of further injury, which could exclude athletes from training and competing for a longer time [12].

Volleyball is one of the most popular sports in the world and is often chosen by amateurs [13]. It requires dynamic changes of direction and movement on a small court [14]. Jumping and falling, in particular, predispose one to back pain, and back injuries are common among volleyball players [15]. Due to those risks, the issue of functional diagnosis in athletes is becoming increasingly important [14]. A commonly used screening test for this purpose is the Functional Movement Screen (FMS), designed for functional assessment [16]. The FMS is a concept that can diagnose deficits in mobility, stability, and proprioception that may lead to injury [17,18]. The FMS consists of seven exercises, each worth a maximum of 21 points. It allows for the prediction of injury risk and is a useful tool for physiotherapists to guide manual therapy and exercise. It provides a comprehensive picture of the athlete and their motor skills [18].

In this study, we assessed the prevalence and severity of back pain among recreational volleyball players. The primary objective is to assess whether FMS test results differ between volleyball players with and without mild back pain. The hypothesis is that the FMS test results are worse among volleyball players with back pain, even with a mild course.

## 2. Materials and Methods

### 2.1. Study Design

This is a cross-sectional study, and we reported the protocol according to STROBE guidelines (the checklist is attached in Supplementary Material S1) [19]. The Bioethical Committee of the Medical University of Warsaw approved this study (AF#075550 from 11 April 2022). Participants received written information about the study purposes and provided their informed consent. All the study procedures were in line with the Declaration of Helsinki.

The recruitment period started on 11 April 2022 and ended on 5 May 2022. We implemented voluntary enrollment and convenience sampling. The participants were invited through the advertisements on an online portal dedicated to amateur volleyball players, PiłkaNaHali.pl (https://www.pilkanahali.pl/; accessed on 11 April 2022), and during three amateur sporting events: the Białołęka Volleyball League (https://ligasiatkowki.pl; accessed on 11 April 2022), the Ursus Volleyball League (https://liga-ursus.siatkowka.org; accessed on 11 April 2022), and the 15th Night Volleyball Tournament.

**Figure 1:**
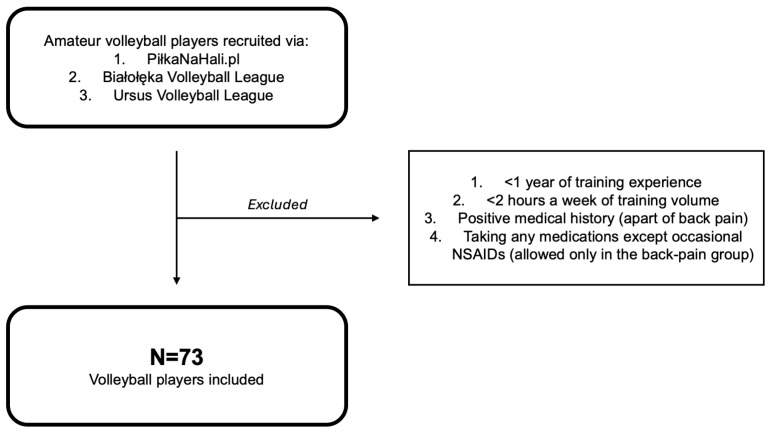
Flow diagram of the recruitment process. Abbreviations: NSAID, non-steroidal anti-inflammatory drug.

Inclusion criteria were as follows: (1) have at least 1 year of experience in volleyball, (2) practice volleyball at least 2 h per week, (3) deny any medical conditions apart from back pain, and (4) not take medications on an ad hoc basis or chronically (participants who reported back pain could only use NSAIDs, and no other medications were allowed). Healthy reference subjects in this study did not take any medications (including NSAIDs). Not fulfilling any of the inclusion criteria was considered an exclusion criterion. The assessment of inclusion and exclusion criteria was carried out by the supervisor during recruitment. All the participants were amateur volleyball athletes. To define the term “Athlete”, we adjusted the Bethesda Conference guidelines: “*one who participates in an organized team or individual sport that requires regular competition against others as a central component, places a high premium on excellence and achievement, and requires some form of systematic (and usually intense) training*” [20]. Because the participants were amateur athletes, they did not compete in professional competitions. They trained regularly but did not receive any compensation or gratification for their athletic achievements. The flow diagram of the recruitment process is presented in Figure 1.

### 2.2. Initial Classification Protocol and the Oswestry Disability Index

Firstly, the participants completed the 10-point author’s questionnaire regarding general demographic information, training experience, degree of back pain according to the Visual Analog Scale, and possible duration of symptoms. The questionnaire transcript in English can be found in Supplementary Material S2 (Part 1). Based on the reported back pain, athletes were classified into two groups: with back pain and without back pain.

Participants classified as having back pain then completed the Oswestry Disability Index (ODI) questionnaire. The ODI questionnaire is tailored for individuals with back pain. We did not require pain-free players to complete the questionnaire. The English transcription of the ODI questionnaire and the grading scheme used in our study are provided in Supplementary Material S2 (Part 2). The questionnaire is one of the most popular tools used for screening people with back pain and has several validation studies [21]. The ODI consisted of 10 questions; each one is graded from 0 (lack of disability) to 5 (severe disability). The final score is the sum of the scores for the 10 questions, and the maximum score is 50 points. Questions are related to (1) pain intensity, (2) grooming, (3) walking, (4) sitting, (5) standing, (6) sleeping, (7) social life, (8) travelling, and (9) change in pain intensity. The % of disability score is the sum of points from each section divided by 50. Results in the range of 0–20% indicate a minimal disability, 21–40% indicate moderate disability, 41–60% indicate severe disability, 61–80% indicate crippled, and 81–100% indicate bedbound [21].

### 2.3. Functional Movement Screen Test

Finally, all participants underwent a functional assessment using the FMS test, as recommended by the methodology [16]. All the FMS tests took place in the Centre of Sport and Rehabilitation of the Medical University of Warsaw (Medical University of Warsaw, Warsaw, Poland). Participants were introduced to the protocol and familiarized with the exercises. During exercises, they were supervised by a qualified exercise physiologist with experience in the assessment of FMS to avoid biased results. Each participant received visual demonstration instructions and oral instructions to perform the FMS correctly. The FMS was conducted according to a protocol consisting of seven exercises. A maximum score of 21 points was possible. Each exercise was performed three times and then rated on a scale of 0 to 3. Exercise #1 is a deep squat with the pole overhead. Exercise #2 is a hurdle step. Exercise #3 is an inline lunge, which involves performing a squat in a lunge position with a pole. Exercise #4 tests shoulder girdle mobility. Exercise #5 tests the active straight-leg raise. Exercise #6 is a trunk stability push-up. The starting position is a front lying position. Exercise #7 is a rotary stability test to test trunk stability in the sagittal plane during upper and lower limb movements.

### 2.4. Study Population

We recruited a total of 79 individuals, including 23 (29.1%) females and 56 (70.9%) males. Males had higher height (185.7 ± 7.2 cm vs. 172.3 ± 6.7 cm), weight (86.9 ± 12.9 kg vs. 68.7 ± 9.2 kg), and BMI (25.1 ± 2.8 kg/m^2^ vs. 23.1 ± 3.0 kg/m^2^) than females. Average age was 28.9 ± 6.9 for males and 28.5 ± 5.2 for females. Males also have higher training experience than females (11.1 ± 7.2 years vs. 10.8 ± 5.8 years). However, females reported higher pain during the strongest episode of back pain in their life according to the VAS scale (5.6 ± 3.0 vs. 5.0 ± 3.2).

In total, 31 (39.2%) athletes reported that they currently experienced back pain, and 67 (84.8%) reported that they had ever experienced back pain regardless of the degree. We present the comparative data between males and females in Table 1 for demographic data and in Table 2 for FMS scores. The overall FMS score did not differ significantly between sexes (14.38 ± 2.69 vs. 13.52 ± 3.57; t(77) = −1.16; *p* = 0.25). However, males have a lower score in active straight-leg raise (1.79 ± 0.78 vs. 2.35 ± 0.78; t(77) = 2.92; *p* = 0.005; *p*_adjusted_ = 0.04) and a higher score in trunk stability push-up (2.50 ± 0.76 vs. 1.09 ± 1.16; t(77) = −6.37; *p* < 0.001; *p*_adjusted_ < 0.005)**.** Therefore, we conducted further comparisons independently for both sexes.

### 2.5. Statistical Analysis

We assessed the normality of the distribution using the Shapiro–Wilk test and Q-Q plots. Because the distribution was normal, continuous data were presented as the mean with standard deviation. Categorical data are presented numerically and in percentages (%). There were no missing data, and therefore, there was no necessity to apply imputation methods.

We assessed the population size in G*Power (V.3.1) post hoc for large effect size (Cohen’s d > 0.8) for all statistical tests used [22,23]. To compare the mean FMS scores between women and men with and without back pain, we used the Student *t*-test for independent means. There were the following subgroups in the analysis: males (*n* = 56; with back pain [*n* = 37] and without back pain [*n* = 19]), females (*n* = 23; with back pain [*n* = 12] and without back pain [*n* = 11]). A sample of *n* = 56 has a power of 0.795 to detect results with a large effect size (d = 0.8). A sample of *n* = 23 has a power of 0.448 to detect results with a large effect size (d = 0.8).

We considered *p*-value < 0.05 as significant. Because multiple comparisons were made in the FMS test (7 exercises and total score; in summary, 8 comparisons), we applied the Bonferroni Correction. We obtained an adjusted significance level of 0.00625 (0.05/8).

We performed analyses in the SPSS Software (V.30.0.0; IBM Corp., Armonk, NY, USA) and we derived the plots in the Graph-Pad Prism (v10.0.0; GraphPad Software, Boston, MA, USA). We present data according to the 11th edition of the *AMA Manual of Style* guidelines.

## 3. Results

### 3.1. Severity of Back Pain Among Volleyball Players

There were 19 (61.3%) males and 12 (38.7%) females with ongoing back pain. Table 3 reports the results of the ODI questionnaire stratified by each question. Only participants who reported ongoing back pain (*n* = 31; 39.2%) completed the ODI questionnaire. The population achieved a score of 3.9 ± 6.1 in ODI, which indicates mild dysfunction. Briefly, 11 (35.5%) participants had scores between 21 and 40% and 2 (6.5%) participants had scores between 41 and 60%. The remaining players had scores below or equal to 20%.

### 3.2. Results of the FMS Stratified by the Back Pain

**Figure 2:**
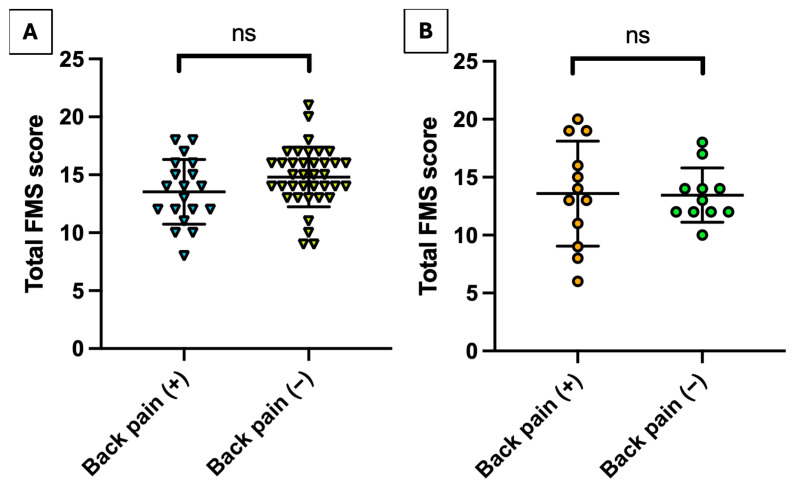
Total FMS scores. Note: Panel (**A**) presents scores for males, and Panel (**B**) for females. (+) indicates present back pain and (−) indicates lack of ongoing back pain. The central bar presents the mean, and the upper/lower bars present the standard deviations. Abbreviations: FMS, Functional Movement Screen; ns, not significant.

Table 4 presents a comparative analysis of the FMS scores obtained by males and females with and without back pain. There were no significant differences between males who reported their back pain and healthy reference controls (*p* = 0.07–0.97). The same relationship occurred among females (*p* = 0.30–0.90). Interestingly, only one female achieved a score = 0 in the rotary stability exercise, and all the remaining females had scores =2. Total FMS scores were not influenced by the occurrence of back pain, both among males (14.81 ± 2.57 vs. 13.53 ± 2.80; t(54) = 1.72, *p* = 0.09) and females (13.58 ± 4.52 vs. 13.46 ± 2.34; t(21) = −0.09, *p* = 0.93). We have shown the relationship in total FMS scores in Figure 2 for males (panel A) and females (panel B). After application of the Bonferroni Correction, the results were even less significant (*p*_adjusted_ = 0.56–1.00).

## 4. Discussion

Briefly, in this study, we confirmed that FMS scores are comparable between participants with back pain and healthy reference controls. Moreover, we showed that back pain is a prevalent medical condition among volleyball players on a recreational level. We also underscored that most athletes rated their back pain as mild, and the functional dysfunction was not severe. The key message from our study highlights the need for preventive strategies among volleyball players regardless of whether they report back pain or not.

Injuries are a significant problem among professional volleyball players [15]. Retrospective analysis of 4-year data from the FIVB Injury Surveillance System confirmed that there were 3.8 injuries per 1000 h of play [24]. Overuse injuries (like back pain) are less common than acute injuries (like ankle sprain) [25]. However, there were no significant differences in the injury rate between males and females. The most injured areas were the ankle (25.9%), knee (15.2%), fingers (10.7%), and lower back (8.9%). The injury rate depended on the position on the court, with middle hitters being most at risk and liberos being least at risk [26]. Therefore, training should include accessory work to minimize the risk of severe back injuries [24].

Our volleyball players had only mild back pain, and there were no significant differences in FMS scores between those with and without back pain. This may potentially be due to the relatively long reported training experience, which was >10 years in both sexes. Mizoguchi and colleagues underscored the link between back pain prevalence and training experience [27]. The authors observed a higher incidence of back pain among volleyball players with more training experience. However, in their study, the players had approximately 3–4 years of training experience and were elite players. Therefore, this relationship may be slightly different in our study.

In our study, lifetime back pain was a very common medical condition, with nearly 85% having experienced back pain. We supplemented the knowledge presented by Skazalski and colleagues here, as they indicated that the in-season prevalence of back pain is as high as 71% [28]. However, their study included elite athletes, while ours included amateurs. Therefore, we note that back pain is very common in volleyball regardless of the level.

It is also worth assessing in future research how back pain affects proprioception and dynamic stability in volleyball players. There are premises that other injuries (e.g., shoulder) aggravate these indicators [29]. In some disciplines, even sex could influence them [30]. However, the impact of back pain in volleyball players requires further investigation. We assessed the overall prevalence and severity of back pain. Other studies have indicated that player position influences back pain [27,29,31]. It would be interesting to conduct an FMS test and assess movement quality in volleyball players stratified by playing position.

Although volleyball is less injury-prone than other team sports (e.g., soccer), volleyball players are still susceptible to numerous injuries due to the specific nature of the sport [24,32]. Numerous jumps, landings, and changes of direction predispose them to common mechanical injuries [24,25]. Our study showed that FMS test results were slightly worse among athletes who reported back pain. This indicates that athletes with back pain have a lower overall functional assessment and a higher risk of other injuries in the future. Our results are consistent with previous work showing that injuries among volleyball players are generally mild and back pain rarely eliminates them from training or matches [24]. Most participants described their disabilities as mild, and FMS did not differ significantly. Therefore, all the participants, regardless of pain, could continue their training, and their functional assessment was not impaired.

We observed no significant differences between men and women in FMS scores for all exercises except the active straight-leg raise and trunk stability push-up. Women generally have lower functional scores. Anderson and colleagues also noted significant differences in FMS scores for the trunk stability push-up, but not for the active straight-leg raise [33]. This does not change the fact that women should pay more attention to movement quality. We emphasize that our study has limited utility in predicting future injuries, primarily indicating slightly limited functional abilities among volleyball players. We used the FMS test, which can be used to predict injuries, but its effectiveness is limited [34]. We observed no significant differences between individuals with and without back pain. Therefore, FMS is a poor distinguishing method. We also recommend that FMS be used to assess movement quality, not prediction of injury risk or severity of impairment [35].

Our results show that there is a need for implementation of prevention and treatment strategies for back pain injuries among volleyball players [24,36]. Accessory exercise programs have a reliable and well-proven use in reducing the risk of back pain in volleyball players [31]. Preferred approaches include load reduction, proper warm-up, correcting imbalances, and strengthening core muscles [37]. Future research should consider categorizing athletes into specific age groups and specific sports disciplines. Individual sports differ in their movement patterns, which means that both training and research should be tailored to their specific characteristics [38,39].

### Limitations

Our study has some limitations. We implemented convenience sampling with the post hoc analysis of the power of our sample. Ideally, we should establish the required power before starting the recruitment, and this is the point for further studies on that topic. We based our analysis on declarative data about the presence and severity of back pain. Some athletes, more habituated to pain and discomfort, could declare lower results, while more sensitive athletes, especially females, could declare more severe pain. There were also a few overweight participants, and it is well known that excess body weight aggravates the risk of back pain. Moreover, participants were amateur athletes, and we did not check their daily life habits (e.g., sitting in a bad position during work, etc.). Bad daily habits could influence the posture and aggravate the back pain. FMS is also assessed by the supervising physiologist. The limited predictive role of FMS should also be considered. Typically, the FMS could accurately predict about half of all injuries [40]. In our study, results were assessed by an experienced exercise physiologist; however, we cannot fully diminish the risk of subjective assessment. Previous studies indicated that injury type could be influenced by the playing position. We analyzed the data independently for males and females. Males achieved power on the verge of significance (79.5%), and females achieved power of 44.8%. Both powers were slightly too low and should optimally be at 80%. We did not assess the playing position and. asked only about the general practice in volleyball. Finally, typical limitations for cross-sectional studies occur, such as the inability to assess the causal relationships. Therefore, we recommend interpreting the results carefully.

## 5. Conclusions

There are no differences in FMS scores in volleyball players with and without back pain, both among males and females. Back pain is a common medical condition and most often has a mild course among recreational volleyball players. Volleyball players with back pain have comparable FMS results to healthy reference athletes. Despite no functional score differences, the high prevalence of back pain suggests the need for prevention.

## Figures and Tables

**Table 1:** Characteristics of the study population.

Sex	Demographic Characteristics					
	Age (Years)	Height (cm)	Weight (kg)	BMI (kg/m^2^)	Training Experience (Years)	Strongest Episode of Back Pain (VAS)
**Males (*n* = 56, 70.9%)**	28.9 ± 6.9	185.7 ± 7.2	86.9 ± 12.9	25.1 ± 2.8	11.1 ± 7.2	5.0 ± 3.2
**Females (*n* = 23, 29.1%)**	28.5 ± 5.2	172.3 ± 6.7	68.7 ± 9.2	23.1 ± 3.0	10.8 ± 5.8	5.6 ± 3.0

Abbreviations: BMI, body mass index; VAS, Visual Analog Scale. Note: Continuous data are presented as mean with standard deviation (±), and categorical data are presented as number and percentage (%).

**Table 2:** Comparison of the FMS scores between males and females.

Sex	FMS							
	#1|Deep Squat	#2|Hurdle Step	#3| Inline Lunge	#4|Shoulder Girdle Mobility	#5|Active Straight-Leg Raise	#6|Trunk Stability Push-Up	#7|Rotary Stability	Total Score
**Males** **(*n* = 56, 70.9%)**	1.96 ± 0.87	2.23 ± 0.83	2.41 ± 0.73	1.52 ± 1.01	1.79 ± 0.78	2.50 ± 0.76	1.96 ± 0.27	14.38 ± 2.69
**Females** **(*n* = 23, 29.1%)**	1.61 ± 0.99	2.26 ± 0.54	2.30 ± 1.06	2.00 ± 1.04	2.35 ± 0.78	1.09 ± 1.16	1.91 ± 0.42	13.52 ± 3.57
***t*-test (df)**	−1.58 (77)	0.15 (77)	−0.51	1.91 (77)	2.92 (77)	−6.37 (77)	−0.65 (77)	−1.16 (77)
***p*-value**	0.12	0.88	0.61	0.06	0.005	<0.001	0.52	0.25
**Adjusted *p*-value**	0.96	1.00	1.00	0.48	0.04	<0.005	1.00	1.00

Abbreviations: #, number; FMS, Functional Movement Screen; df, degrees of freedom. Note: Differences between sexes were calculated with the Student *t*-test. Continuous data are presented as mean with standard deviation (±), and categorical data are presented as number and percentage (%). Due to there being 8 multiple comparisons, we applied the Bonferroni Correction, and we presented the adjusted *p*-values.

**Table 3:** Results of the ODI questionnaire.

Question	#1	#2	#3	#4	#5	#6	#7	#8	#9	#10	Total Score
**Score**	2.1 ± 1.3	0.9 ± 0.9	0.7 ± 0.9	0.2 ± 0.7	1.0 ± 1.1	1.3 ± 1.1	0.5 ± 0.6	0.3 ± 0.5	1.0 ± 1.1	1.8 ± 1.4	3.9 ± 6.1

Abbreviations: #, number. Note: Questions are listed in the Supplementary Material S2. Data are presented as mean with standard deviation (±) for each question. Each question could be graded from 0 to 5 based on the severity of disability. The maximal score is 50 points.

**Table 4:** Results of the FMS test.

Exercise	Males (*n* = 56, 70.9%)						Females (*n* = 23, 29.1%)					
	With Back Pain (*n* = 37, 66.1%)	Without Back Pain (*n* = 19, 33.9%)	*t*-Test (df)	ES (95%CI)	*p*-Value	Adjusted *p*-Value	With Back Pain (*n* = 12, 52.2%)	Without Back Pain (*n* = 11, 47.8%%)	*t*-Test (df)	ES (95%CI)	*p*-Value	Adjusted *p*-Value
**#1|deep squat**	1.74 ± 0.99	2.08 ± 0.80	1.41 (54)	0.40 (−0.16, 0.95)	0.16	1.00	1.58 ± 1.31	1.64 ± 0.51	0.13 (21)	0.05 (−0.77, 0.87)	0.90	1.00
**#2|hurdle step**	1.95 ± 1.03	2.39 ± 0.68	1.88 (54)	0.53 (−0.03, 1.09)	0.07	0.56	2.33 ± 0.49	2.18 ± 0.60	−0.66 (21)	−0.28 (−1.10, 0.55)	0.52	1.00
**#3|inline lunge**	2.42 ± 0.61	2.41 ± 0.80	−0.08 (54)	−0.21 (−0.57, 0.53)	0.94	1.00	2.42 ± 0.17	2.18 ± 0.92	−0.52 (21)	−0.22 (−1.04, 0.61)	0.61	1.00
**#4|shoulder girdle mobility**	1.53 ± 1.02	1.51 ± 1.02	−0.05 (54)	−0.13 (−0.57,0.54)	0.97	1.00	1.83 ± 1.03	2.18 ± 1.08	0.79 (21)	0.33 (−0.50, 1.15)	0.44	1.00
**#5|active straight-leg raise**	1.58 ± 0.77	1.89 ± 0.77	1.44 (54)	0.41 (−0.16, 0.96)	0.16	1.00	2.25 ± 0.87	2.46 ± 0.69	0.62 (21)	0.26 (−0.57, 1.08)	0.54	1.00
**#6|trunk stability push-up**	2.37 ± 0.83	2.57 ± 0.73	0.92 (54)	0.26 (−0.30, 0.82)	0.36	1.00	1.33 ± 1.37	0.82 ± 0.87	−1.06 (21)	−0.44 (−1.27, 0.39)	0.30	1.00
**#7|rotary stability**	1.95 ± 0.23	1.97 ± 0.29	0.34 (54)	0.10 (−0.46, 0.65)	0.74	1.00	1.83 ± 0.58	2.00 ± 0.00	N/A	N/A	N/A	N/A
**Total score**	13.53 ± 2.80	14.81 ± 2.57	1.72 (54)	0.49 (−0.08, 1.04)	0.09	0.72	13.58 ± 4.52	13.46 ± 2.34	−0.09 (21)	−0.04 (−0.85, 0.78)	0.93	1.00

Abbreviations: #, number; df, degrees of freedom; ES, effect size; 95%CI, 95% confidence interval; N/A, not applicable. Note: Differences between individuals with and without back pain were calculated with the Student *t*-test. Continuous data are presented as mean with standard deviation (±). For rotary stability among females, only 1 player achieved a score = 0 (in a group with back pain), and all remaining participants had a score = 2. Due to there being 8 multiple comparisons, we applied the Bonferroni Correction, and we presented the adjusted *p*-values.

## Data Availability

The raw data supporting the conclusions of this article will be made available by the authors on request.

## Supplementary Materials

The following supporting information can be downloaded at https://www.mdpi.com/article/10.3390/jcm14186502/s1, Supplementary Material S1: STROBE checklist; Supplementary Material S2: Author’s and ODI questionnaires.
