# Supplementary material for: Functional Movement Screen Scores Are Comparable in Volleyball Players with and Without Back Pain—The FMS-VBP Study
Source: J Clin Med. 2025 Sep 15;14(18):6502. doi: 10.3390/jcm14186502 (PMC12470880; doi:10.3390/jcm14186502)
Supplement: Supplementary file 1 [file jcm-14-06502-s001.zip › jcm-3863555-supplementary.pdf]

Supplementary Material 1. STROBE checklist

STROBE Statement—checklist of items that should be included in reports of observational studies

	Item No	Recommendation	Reported (✓) Not reported (✗)
**Title and abstract**	1	(*a*) Indicate the study's design with a commonly used term in the title or the abstract	✓
		(*b*) Provide in the abstract an informative and balanced summary of what was done and what was found	✓
**Introduction**			
Background/rationale	2	Explain the scientific background and rationale for the investigation being reported	✓
Objectives	3	State specific objectives, including any prespecified hypotheses	✓
**Methods**			
Study design	4	Present key elements of study design early in the paper	✓
Setting	5	Describe the setting, locations, and relevant dates, including periods of recruitment, exposure, follow-up, and data collection	✓
Participants	6	(*a*) *Cohort study*—Give the eligibility criteria, and the sources and methods of selection of participants. Describe methods of follow-up<br>*Case-control study*—Give the eligibility criteria, and the sources and methods of case ascertainment and control selection. Give the rationale for the choice of cases and controls<br>*Cross-sectional study*—Give the eligibility criteria, and the sources and methods of selection of participants	✓
		(*b*) *Cohort study*—For matched studies, give matching criteria and number of exposed and unexposed<br>*Case-control study*—For matched studies, give matching criteria and the number of controls per case	✓
Variables	7	Clearly define all outcomes, exposures, predictors, potential confounders, and effect modifiers. Give diagnostic criteria, if applicable	✓
Data sources/ measurement	8*	For each variable of interest, give sources of data and details of methods of assessment (measurement). Describe comparability of assessment methods if there is more than one group	✓
Bias	9	Describe any efforts to address potential sources of bias	✓
Study size	10	Explain how the study size was arrived at	✓
Quantitative variables	11	Explain how quantitative variables were handled in the analyses. If applicable, describe which groupings were chosen and why	✓
Statistical methods	12	(*a*) Describe all statistical methods, including those used to control for confounding	✓
		(*b*) Describe any methods used to examine subgroups and interactions	✓
		(*c*) Explain how missing data were addressed	✓
		(*d*) *Cohort study*—If applicable, explain how loss to follow-up was addressed<br>*Case-control study*—If applicable, explain how matching of cases and controls was addressed	✓

		Cross-sectional study—If applicable, describe analytical methods taking account of sampling strategy	
		(*e*) Describe any sensitivity analyses	✖
**Results**			
Participants	13*	(a) Report numbers of individuals at each stage of study—eg numbers potentially eligible, examined for eligibility, confirmed eligible, included in the study, completing follow-up, and analysed	✓
		(b) Give reasons for non-participation at each stage	✓
		(c) Consider use of a flow diagram	✖
Descriptive data	14*	(a) Give characteristics of study participants (eg demographic, clinical, social) and information on exposures and potential confounders	✓
		(b) Indicate number of participants with missing data for each variable of interest	✓
		(c) *Cohort study*—Summarise follow-up time (eg, average and total amount)	✓
Outcome data	15*	*Cohort study*—Report numbers of outcome events or summary measures over time	✓
		Case-control study—Report numbers in each exposure category, or summary measures of exposure	✓
		Cross-sectional study—Report numbers of outcome events or summary measures	✓
Main results	16	(*a*) Give unadjusted estimates and, if applicable, confounder-adjusted estimates and their precision (eg, 95% confidence interval). Make clear which confounders were adjusted for and why they were included	✓
		(*b*) Report category boundaries when continuous variables were categorized	✓
		(*c*) If relevant, consider translating estimates of relative risk into absolute risk for a meaningful time period	✓
Other analyses	17	Report other analyses done—eg analyses of subgroups and interactions, and sensitivity analyses	✓
**Discussion**			
Key results	18	Summarise key results with reference to study objectives	✓
Limitations	19	Discuss limitations of the study, taking into account sources of potential bias or imprecision. Discuss both direction and magnitude of any potential bias	✓
Interpretation	20	Give a cautious overall interpretation of results considering objectives, limitations, multiplicity of analyses, results from similar studies, and other relevant evidence	✓
Generalisability	21	Discuss the generalisability (external validity) of the study results	✓
**Other information**			
Funding	22	Give the source of funding and the role of the funders for the present study and, if applicable, for the original study on which the present article is based	✓

*Give information separately for cases and controls in case-control studies and, if applicable, for exposed and unexposed groups in cohort and cross-sectional studies.

Note: An Explanation and Elaboration article discusses each checklist item and gives methodological background and published examples of transparent reporting. The STROBE checklist is best used in conjunction with this article (freely available on the Web sites of PLoS Medicine at http://www.plosmedicine.org/, Annals of Internal Medicine at http://www.annals.org/, and Epidemiology at http://www.epidem.com/). Information on the STROBE Initiative is available at www.strobe-statement.org.

Supplementary Material 2. Author's and ODI questionnaires

Part 1. General and demographic data

English transcript

Question 1. Sex
- Male
- Female

Question 2. Age (insert exact number in years)

Question 3. Weight (insert exact number in kg)

Question 4. Height (insert exact number in cm)

Question 5. How many hours a week do you train volleyball? (insert exact number in hours)

Question 6. How many years have you been training volleyball? (insert exact number in years)

Question 7. Rate your worst back pain episode on a Visual Analog Scale scale from 0 to 10, where 0 is no back pain, 10 is unbearable pain (insert exact number)

Question 8. Rate your current back pain on a Visual Analog Scale scale from 0 to 10, where 0 is no back pain, 10 is unbearable pain (insert exact number)

Question 9. Do you currently experience back pain (select the appropriate answer): YES / NO

Question 10. If you currently experience back pain, please indicate how long your back has been hurting:
- <1 year
- 1-2 years
- 2-3 years
- 3-4 years
- 5-6 years
- 7-8 years
- >8 years

Part 2. Oswestry Disability Index

English transcript

Instructions

Complete this questionnaire if you have had back pain in the last month.
This questionnaire was designed to help you understand how much back pain affects your ability to perform daily activities. Please select only one answer in each section that best describes your functionality.

Question 1. Pain intensity

- The pain is very mild, comes and goes
- The pain is mild, constant
- The pain is moderate, comes and goes
- The pain is moderate and constant
- The pain is severe, comes and goes
- The pain is severe and constant

Question 2. Grooming (washing, dressing, etc.)

- I do not need to change the way I wash or dress to avoid pain
- I usually do not change the way I wash or dress, even though it causes mild pain
- Washing and dressing causes increased pain, but I manage without changing the way I do these activities
- Washing and dressing causes increased pain, which forces me to change the way I do these activities
- Because of the pain, I am unable to perform some washing and dressing activities without assistance
- Because of the pain, I am unable to wash or dress without assistance (5)

Question 3. Lifting

- I can lift heavy objects without pain
- I can lift heavy objects, but lifting causes pain
- Pain prevents me from lifting heavy objects from the floor
- Pain prevents me from lifting heavy objects from the floor, but I can manage if they are conveniently arranged, e.g., on a table
- Pain prevents me from lifting heavy objects, but I can lift light and not too heavy objects if they are conveniently arranged
- I can only lift light objects

Question 4. Walking

- Pain does not prevent me from walking any distance
- Pain prevents me from walking more than 1500 m
- Pain prevents me from walking more than 800 m
- Pain prevents me from walking more than 400 m
- I can only walk with a cane or crutches
- I spend most of my time in bed and have to crawl to the toilet

Question 5. Sitting

- I can sit in any chair as long as I want without pain
- I can only sit in my favorite chair as long as I want
- Pain prevents me from sitting for more than one hour
- Pain prevents me from sitting for more than half an hour
- Pain prevents me from sitting for more than 10 minutes
- Pain prevents me from sitting at all

Question 6. Standing

- I can stand as long as I want without pain
- I have a little pain when I stand, but the pain doesn't get worse
- I can't stand for more than an hour without the pain getting worse
- I can't stand for more than half an hour without the pain getting worse
- I can't stand for more than 10 minutes without the pain getting worse
- I avoid standing because the pain gets worse immediately

Question 7. Sleeping

- I don't feel any pain when I lie in bed
- I feel pain when I lie in bed, but it doesn't interfere with my sleep
- Due to I only sleep 3/4 of the night because of the pain
- I only sleep 1/2 of the night because of the pain
- I only sleep 1/4 of the night because of the pain
- I don't sleep at all because of the pain

Question 8. Social Life

- My social life is normal and doesn't cause me back pain
- My social life is normal, but it increases the pain
- The pain doesn't significantly affect my social life, but I have to avoid more demanding activities like dancing, etc.
- The pain limits my social life and I don't leave the house very often
- Because of the pain, I limit my entire social life to gatherings at home
- Because of the pain, I have almost no social life

Question 9. Traveling

- I don't experience pain when traveling
- I experience some pain when traveling, but none of my usual forms of travel
- increase the pain
- I experience additional pain when traveling, but it doesn't force me to seek other
- ways of travel
- I experience additional pain while traveling, which forces me to seek other
- ways of travel
- The pain limits all forms of travel
- The pain prevents me from traveling unless I can lie down during the trip

Question 10. Change in pain intensity

- My pain is rapidly subsiding
- My pain is fluctuating, but is generally subsiding
- My pain seems to be subsiding, but is currently improving slowly
- My pain is neither subsiding nor increasing

- My pain is gradually increasing
- My pain is rapidly increasing

Thank you for completing the survey. The next step is the exercise tests, which will be administered by the researcher.
